# Femtosecond time-evolution of mid-infrared spectral line shapes of Dirac fermions in topological insulators

**DOI:** 10.1038/s41598-020-66720-4

**Published:** 2020-06-17

**Authors:** Tien-Tien Yeh, Chien-Ming Tu, Wen-Hao Lin, Cheng-Maw Cheng, Wen-Yen Tzeng, Chen-Yu Chang, Hideto Shirai, Takao Fuji, Raman Sankar, Fang-Cheng Chou, Marin M. Gospodinov, Takayoshi Kobayashi, Chih-Wei Luo

**Affiliations:** 1 Department of Electrophysics, National Chiao Tung University, Hsinchu, Taiwan; 2 National Synchrotron Radiation Research Center, Hsinchu, 30076 Taiwan; 3 Institute for Molecular Science, 38 Nishigonaka, Myodaiji, Okazaki, 444-8585 Japan; 4 Toyota Technological Institute, 2-12-1 Hisakata, Tempaku-ku, Nagoya 468-8511 Japan; 5 Institute of Physics, Academia Sinica, Nankang, Taipei, R.O.C 11529 Taiwan; 6 Center for Condensed Matter Sciences, National Taiwan University, Taipei, 10617 Taiwan; 7 Institute of Solid State Physics, Bulgarian Academy of Sciences, 1784 Sofia, EU Bulgaria; 8 Brain science Inspired Life Support Research Center, The University of Electro-Communications, 1-5 1 Chofugaoka, Chofu, Tokyo 182-8585 Japan; 9 Taiwan Consortium of Emergent Crystalline Materials (TCECM), Ministry of Science and Technology, Taipei, Taiwan; 10 Center for Emergent Functional Matter Science, National Chiao Tung University, Hsinchu, 30010 Taiwan

**Keywords:** Topological insulators, Ultrafast photonics

## Abstract

Mid-infrared (MIR) light sources have much potential in the study of Dirac-fermions (DFs) in graphene and topological insulators (TIs) because they have a low photon energy. However, the topological surface state transitions (SSTs) in Dirac cones are veiled by the free carrier absorption (FCA) with same spectral line shape that is always seen in static MIR spectra. Therefore, it is difficult to distinguish the SST from the FCA, especially in TIs. Here, we disclose the abnormal MIR spectrum feature of transient reflectivity changes (Δ*R*/*R*) for the non-equilibrium states in TIs, and further distinguish FCA and spin-momentum locked SST using time-resolved and linearly polarized ultra-broadband MIR spectroscopy with no environmental perturbation. Although both effects produce similar features in the reflection spectra, they produce completely different variations in the Δ*R*/*R* to show their intrinsic ultrafast dynamics.

## Introduction

Time-resolved spectroscopy is important in various fields, such as determining the exotic carrier dynamics of TIs^1–7^. The photon energy (~100 meV) of a MIR is less than the bulk band gap of TIs and has a very different energy to the resonance energy of phonon absorptions. Therefore, MIR light sources are eminently suited to the study of SSTs in topological surface states (TSSs). The existing literature^8–22^ reports the existence of a spectral line shape in the MIR region but there is no clear consensus. The explanation for FCA based on the Drude model has been adapted^11,12,17^, but some studies give conflicting results^14,18^ with considering more resonance factors. SSTs have also been reported^8–22^ but these studies do not clarify the absorption mechanisms for SSTs and FCA using static MIR spectroscopic techniques.

**Figure 1:**
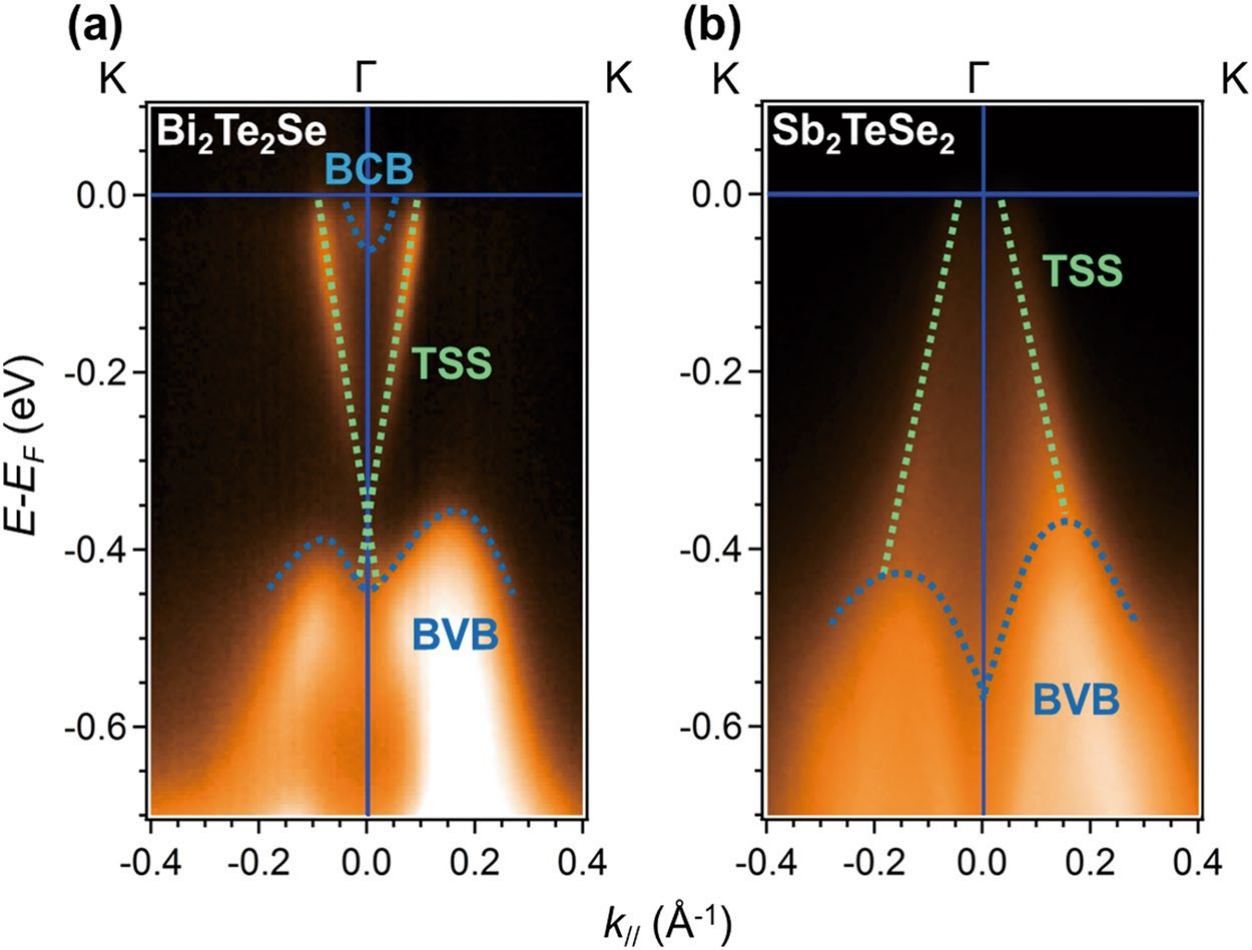
The angle resolved photoemission spectroscopy (ARPES) images of Bi_2_Te_2_Se and Sb_2_TeSe_2_ single crystals: (**a**) The ARPES image of a Bi_2_Te_2_Se single crystal measured with 22 eV photon energy. (**b**) The ARPES image of a Sb_2_TeSe_2_ single crystal measured with 24 eV photon energy. All single crystals were the same pieces as those used in ultrafast experiments for the consistency of all measurements. The single crystals were *in-situ* cleaved under a base pressure 5.1 × 10^−11^ torr at 85 K just before measurements. ARPES experiment was conducted National Synchrotron Radiation Research Center in Taiwan using BL21B1 beamline. The photoemission spectra were recorded with a Scienta R4000 hemispherical analyzer. The polarization vector was always in the angular dispersion plane. The overall energy resolution is about 12 meV. The green dash lines represent as the TSS of crystals, and the blue dash lines show the bulk-conduction-band (BCB) and bulk-valance-band (BVB). The Dirac point in Sb_2_TeSe_2_ was estimated at 189 meV above the Fermi level (see S1 in Supplementary information). A notable difference of band structure exists between Bi_2_Te_2_Se and Sb_2_TeSe_2_, the Dirac point of Bi_2_Te_2_Se is embedded in the BVB. In contrast to Bi_2_Te_2_Se, Sb_2_TeSe_2_ has an isolated Dirac cone and surface carriers cannot be scattered easily by bulk carriers. This difference in their band structure makes a significant difference in optical measurement results.

This study unambiguously demonstrates the time evolution of MIR spectral line shapes in TIs using an optical pump and ultra-broadband MIR probe spectroscopy^23^. The MIR probe-pulses with a supercontinuum of 200–5000 cm^−1^ (or 25–620 meV) and a pulse width of 8.2 fs are generated using four-wave different-frequency generation (DFG) in nitrogen gas. *This novel spectroscopy technique has the advantages of a wide bandwidth for standard Fourier-transform-infrared spectroscopy* (*FTIR)*^24^ *and it allows femtosecond time-resolution by generating ultrashort pulses from nonlinear crystals using DFG*. Two types of TI crystals are used for the experiments in this study. One is n-type Bi_2_Te_2_Se with a bulk/surface carrier concentration of 12.5 × 10^18^ cm^−3^/5.5 × 10^12^ cm^−2^ (see S1 in Supplementary information), which is a bulk-conduction-electron-rich crystal. The other is p-type Sb_2_TeSe_2_ with a bulk/surface carrier concentration of 4.8 × 10^18^ cm^−3^/2.2 × 10^12^ cm^−2^ (see S1 in Supplementary information), which features a higher ratio of surface to bulk carrier concentration. Figure 1 shows the clear presence of a bulk-conduction band (BCB) in Bi_2_Te_2_Se, but not in Sb_2_TeSe_2_.

## Results

### Ultra-broadband MIR Δ*R*/*R* spectra of FCA and SSTs in topological insulators

**Figure 2:**
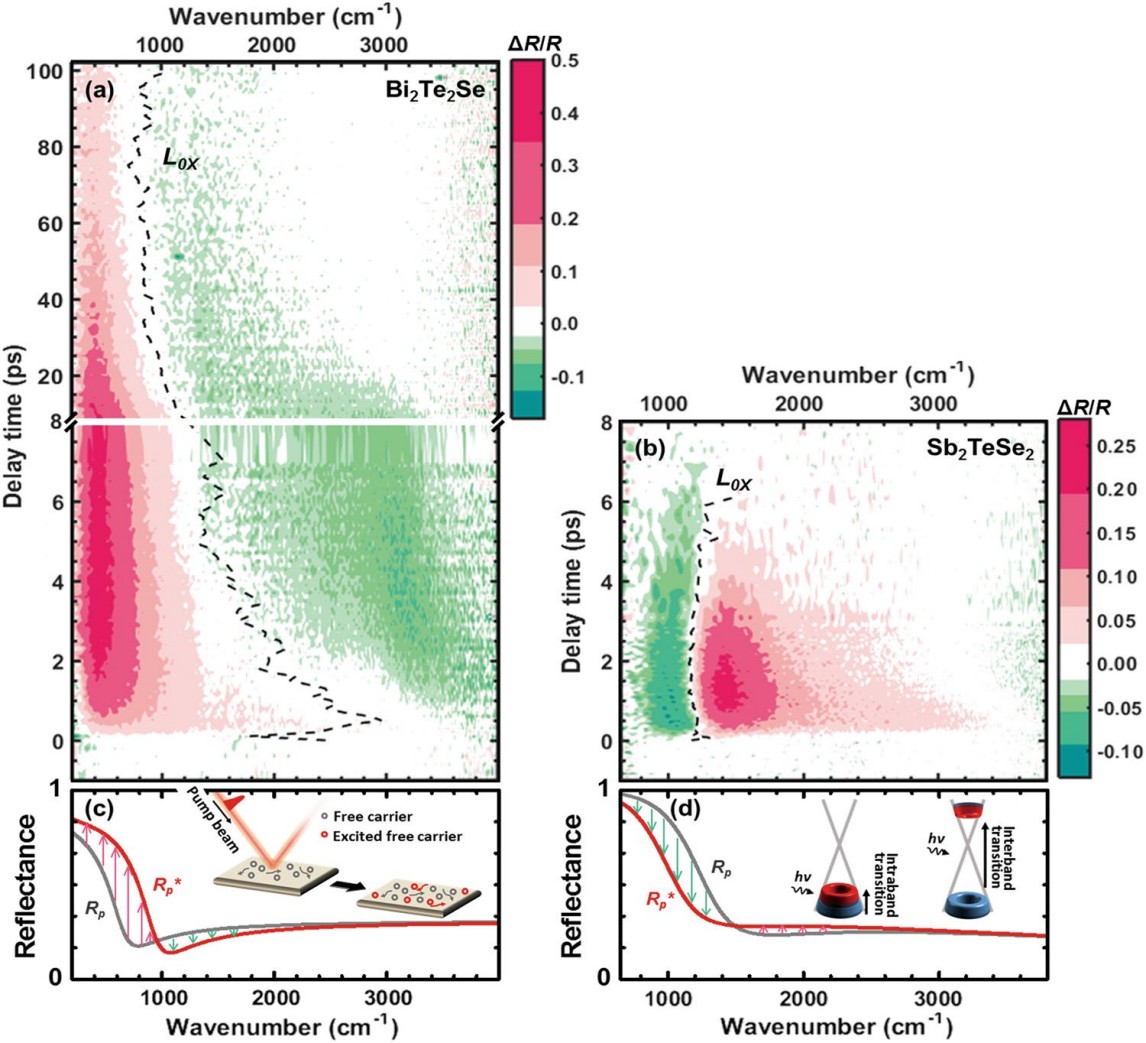
The time-resolved ultra-broadband MIR Δ*R*/*R* spectra for Bi_2_Te_2_Se and Sb_2_TeSe_2_ single crystals and the schematics of the theoretical model: (**a**) and (**b**) the 2D plots of wavenumber- and time-resolved reflectance change (Δ*R*/*R*) spectra with an optical pump fluence of 101 μJ/cm^2^ for Bi_2_Te_2_Se (**a**) and Sb_2_TeSe_2_ (**b**) single crystals. The red and green colors respectively represent the parts with a positive change and a negative change. The zero-crossing line is marked *L*_0*×*_ as a black dashed line. (**c**) shows the p-polarized reflectivity before pumping (*R*_*p*_, gray solid-line. Assume *N* is 12.5 × 10^18^ cm^−1^, so *ω*_*p*_ = 1880 cm^−1^ with m^*^ = 0.32 and *ε*_0_ = 23.7) and after pumping (*R*_p_^*^, red solid-line. Assume *N* is 25 × 10^18^ cm^−1^ so *ω*_*p*_ = 2630 cm^−1^ with m^*^ = 0.32 and *ε*_*0*_ = 23.7) for the Drude model and (**d**) shows the p-polarized reflectivity before pumping (*R*_*p*_, gray solid-line. Assuming *μ* = 50 meV at room temperature) and after pumping (*R*_p_^*^, red solid-line. Assuming *μ* = 40 meV at room temperature) for the SST-Kubo model.

The typical ultra-broadband MIR Δ*R*/*R* spectra for Bi_2_Te_2_Se and Sb_2_TeSe_2_ are respectively shown in Fig. 2a,b. These two spectra are significantly different. Along the wavenumber axis, there is a positive change in the lower frequency region and a negative change in the high frequency region, which indicates a blue-shift in the plasma edge for Bi_2_Te_2_Se after pumping (see Fig. 2c). The zero-crossing line, *L*_0,X_ (dashed line), in Fig. 2a also shows a rapid blue-shift at the beginning of the delay time and then slowly (>50 ps) returns to the original position. However, the value of Δ*R*/*R* for Sb_2_TeSe_2_ shows a red-shift in the plasma edge after pumping. It is worthy of note that the zero-crossing line, *L*_0,X_ (dashed line) in Fig. 2b is red-shifted until ~2 ps and then returns to the original position at ~6 ps, which is much faster than the change for Bi_2_Te_2_Se. Generally, there is a blue-shift in the plasma edge because there is an increase in the carrier concentration^25^, which is explained by the Drude model. The red-shift in the Δ*R*/*R* spectrum of Sb_2_TeSe_2_ until ~2 ps is not explained by the Drude model because there is a decrease in the carrier concentration after pumping. It is found that the SST model using Kubo formula^20^ (SST-Kubo model), which has been successfully used to explain the transitions of Dirac cone in graphene^20^, explains the novel phenomena that are observed in p-type Sb_2_TeSe_2_.

By comparing the band mapping results of Bi_2_Te_2_Se and Sb_2_TeSe_2_ in Fig. 1, a notable difference between Bi_2_Te_2_Se and Sb_2_TeSe_2_ can be found that the Dirac point of Bi_2_Te_2_Se is embedded in the BVB. The surface carriers cannot avoid scattering from bulk carriers, and the major change of optical property might be dominated by bulk carrier. In contrast to Bi_2_Te_2_Se, Sb_2_TeSe_2_ has an isolated Dirac cone and thus the surface carriers cannot be scattered easily by bulk carriers, that is why the SST is a major factor in Sb_2_TeSe_2_. Besides, the difference between bulk FCA of the Bi_2_Te_2_Se and SST of Sb_2_TeSe_2_ could be attributed to the intrinsic responses with a 1.55-eV excitation. As the schematics of Fig. 4e,j, the final and initial states of excitation process are different. The photoexcited carriers of the former are excited from the valence band maximum to the second conduction band^2,4,26^, which is far from the Fermi level. For the latter case, the photoexcited carriers are excited from a deep valance band to the states near Fermi level consisted of an isolated Dirac cone^5^. Therefore, the MIR probe beam tends to detect the free carriers of conduction band in Bi_2_Te_2_Se, and the SST near Fermi level in Sb_2_TeSe_2_.

### Quantitative analysis of the ultra-broadband MIR Δ*R*/*R* spectra

To quantitatively reveal the hidden mechanism, the Drude model and the SST-Kubo model are used to fit the ultra-broadband MIR Δ*R*/*R* spectra for n-type Bi_2_Te_2_Se and p-type Sb_2_TeSe_2_ TIs. It is initially assumed that before and after pumping, all reflectivity *R*_*p*_ (gray solid-line, before pumping) and *R*_p_^*^ (red solid-line, after pumping) have similarly shaped spectra for both the Drude model (Fig. 2c) and the SST-Kubo model (Fig. 2d). After pumping, the reflection spectrum shifts because there is an increase in the free carrier concentration. In terms of the Drude model, the dielectric function *ε*_*D*_ is:

1 $${\varepsilon }_{D}(\omega )={\varepsilon }_{\infty }-\frac{{\omega }_{p}^{2}}{{\omega }^{2}+i\varGamma \omega }$$

where *ε*_*∞*_ is the permittivity at an infinite frequency, *ω* is the frequency, *ω*_*p*_ is the plasma frequency and *Γ* is the plasma scattering rate. The carrier concentration *N* is related to the effective mass m^*^ by the equation, *N* = m^*^ω_*p*_^2^/4*πe*^2^. However, Falkovsky *et al*. estimated the reflectivity by considering the SSTs^20^. The dielectric function using the Kubo formula is:

2 $$\begin{array}{ccc}{\varepsilon }_{F}(\omega ) & = & -\frac{8T}{({\omega }^{2}+i{\tau }^{-1}\omega )\cdot {d}_{TSS}}\left(\frac{{e}^{2}}{\hslash }\right)\mathrm{ln}\,\left[2\,\cosh \left(\frac{\mu }{2T}\right)\right]\\  &  & +\frac{1}{{d}_{TSS}}\left(\frac{{e}^{2}}{\hslash }\right)\,\left[\frac{i\pi }{\omega }G\left(\frac{\omega }{2}\right)-4{\int }_{0}^{+\infty }d\xi \frac{G(\xi )-G\left(\frac{\omega }{2}\right)}{{\omega }^{2}-4{\xi }^{2}}\right]\end{array}$$

where *μ* is the chemical potential, *T* is the carrier temperature, *G* is the Fermi-Dirac distribution function, *τ*^−1^ is the collision rate for TSSs, which depends on the density of impurities, and *d*_*TSS*_ is the optical penetration depth of the TSSs. The first and second terms respectively represent the intra-band transitions and the inter-band transitions in Dirac cone. Both models are applied under the “quasi-equilibrium” state in a view of sub-10 fs probe pulse (see S2 of Supplementary information). The penetration depth of ultra-broadband MIR in TIs is few μm (see S3 of Supplementary information).

As previously mentioned, the increase of *N* in the Drude model represents the change in the electronic population after pumping. In Fig. 2c, the estimated value of *N* for *R*_p_^*^ is larger than that for *R*_*p*_, which results in a blue-shift in the plasma edge. In the SST-Kubo model, the photo-excitation has a significant impact on *μ* and *T* and induces changes in the reflection spectrum. In terms of the ground state of p-type Sb_2_TeSe_2_, both the smaller number of carriers in the vicinity of the Dirac point and the higher electron temperature result in a reduction in *μ*^20^. Therefore, after pumping, the reduction in the chemical potential *μ* causes a change in the reflection spectrum from *R*_*p*_ to *R*_p_^*^, as shown in Fig. 2d. This result is in good qualitative agreement with the Δ*R*/*R* spectrum in Fig. 2b.

### Ultrafast time-evolution of the ultra-broadband MIR Δ*R*/*R* spectra

**Figure 3:**
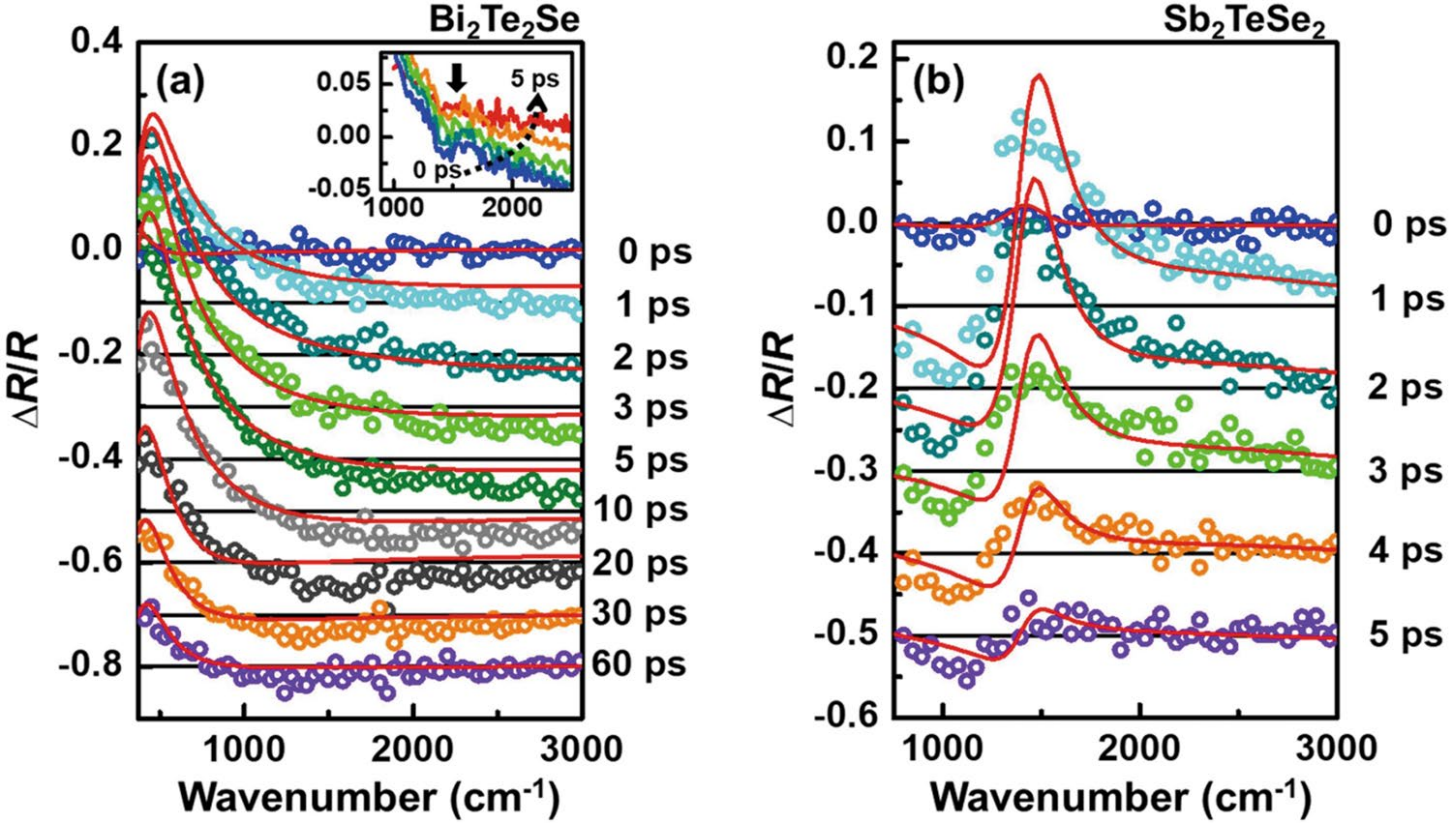
Typical ultra-broadband MIR Δ*R*/*R* spectra with fitting curves, taking account of free carrier absorption (FCA) and surface state transition (SST). Δ*R*/*R* as a function of the wavenumber for different delay times for (**a**) Bi_2_Te_2_Se and (**b**) Sb_2_TeSe_2_, using a pump fluence of 101  μJ/cm^2^. The open circles represent the experimental data and each Δ*R*/*R* spectrum is shifted for clarity. The red lines are the fitting curves with **a** the Drude model and **b** the Drude-SST-Kubo model (*δ*_*ω*_ = −690 cm^−1^). The insert in a shows the Moss-Burstien shift, as indicated by the arrow. Between the top solid line and the bottom solid line are the spectra that are respectively obtained by averaging the data from 0–1 (red), 1–2 (orange), 2–3 (bright green), 3–4 (green) and 4–5 (blue) ps.

Figure 3 shows the typical time-evolution of the MIR Δ*R*/*R* spectrum and the fitted curves. As mentioned previously, the photoexcited carrier dynamics in n-type Bi_2_Te_2_Se is dominated by FCA and can be fitted well with the Drude model, as shown in Fig. 3a. For Sb_2_TeSe_2_, the contribution of FCA to the photoexcited carrier dynamics cannot be neglected. Therefore, the Δ*R*/*R* spectra are fitted with the modified dielectric function of ${\varepsilon }_{DF}(\omega +\delta \omega )={\varepsilon }_{D}(\omega +\delta \omega )+{\varepsilon }_{F}(\omega +\delta \omega )$, where *δ*_*ω*_ is a shifted frequency in fitting (This is called the Drude-SST-Kubo model). Figure 3b shows that this model fits the MIR Δ*R*/*R* spectrum at various delay times quite well. The details of the fitting are presented in the Method section.

## Discussion

**Figure 4:**
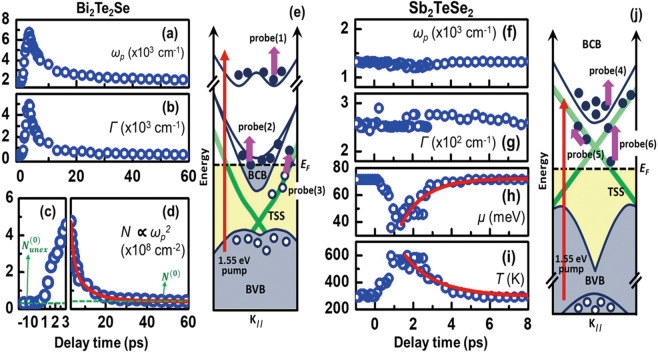
The time-evolution of *ω*_*p*,_ *Γ*, *μ*, *T* and the schematic energy band structure of TIs for pump/probe processes. The fitting results (**a**–**d**) and the pump-probe scheme (**e**) for Bi_2_Te_2_Se and (**f**–**i**) and for Sb_2_TeSe_2_ (**j**). (**a**,**b**) respectively show the time-evolution of the fitting parameter *ω*_*p*_ and *Γ* for the Drude model for a Bi_2_Te_2_Se crystal. (**c**,**d**) show the partial trace of the squared values of *ω*_*p*_ before **c** and after 3 ps (**d**) The red line in **d** shows the bi-exponential fitting that is described in the Method section. The green dashed lines in **c** marked by ${N}_{unex}^{(0)}$ (3.42 × 10^7^ cm^−2^) relate to the concentration of unexcited carriers and (**d**) marked by *N*^(0)^ (4.16 × 10^7^ cm^−2^) represent the height of the constant term from the fitting curve. The time-domain traces for (**f**) *ω*_*p*,_ (**g**) *Γ*, (**h**) *μ* and (**i**) carrier temperature *T* are obtained using the Drude-SST-Kubo model. The red lines in (**h**), (**i**) show the single-exponential fitting that is mentioned in the Method section. The red arrows and pink arrows respectively show the 1.55 eV (800 nm) pumping and the MIR probing in (**e**), (**j**). The notation *E*_*F*_ in (**e**), (**j**) is the Fermi energy.

The fitting results in Fig. 4a,b are of interest, in particular the time evolutions of *ω*_*p*_, *Γ*, *N*, *μ*, and *T* in TI’s. During the pumping process, the 1.55-eV pump photons excite the electrons to a higher BCB from the occupied states^1^. For Bi_2_Te_2_Se, both *ω*_*p*_ and *Γ* respectively exhibit growth and relaxation dynamics. Although it is difficult to obtain the real value of *N* because there is no m^*^, it is still possible to obtain the temporal evolution of *N* through $N\propto {\omega }_{p}^{2}$, as shown in Fig. 4c,d. The seriously shift of *ω*_*p*_ (~3.7 times after photo-excitation) equivalents to the dramatic enhance of photo-excited concentration (see S4 in Supplementary information). This photoexcited carrier mainly experiences FCA in bulk states (BSs), as shown by the notation of probe(1) and probe(2) in Fig. 4e, or in TSSs, as shown by the notation of probe(3). A bi-exponential decay function is further used to obtain the reduction times for the concentration of photoexcited carriers. This has a maximum within ~2.2 ps and then undergoes two relaxation processes for 1.5 ps and 8.4 ps. The fast relaxation process is caused by the thermal diffusion in BCB and TSS^27,28^, or the acoustic-phono assistant process^3^. The slow one is consistent with the results of time-resolved ARPES^1,2^. Additionally, the appearance of a Moss-Burstien shift (until ~6 ps) near the bulk band gap (see the inset in Fig. 3a) also indicates the recombination in BSs^15^. However, the value of *N* (*i.e*., *N*^(0)^ in Fig. 4d) does not recover to its original value (*i.e*., ${N}_{unex}^{(0)}$ in Fig. 4c) within the limited delay time (~ 100 ps). This inconsistency between *N*^(0)^ and ${N}_{unex}^{(0)}$ is explained by the long-lived recombination process (see S5 in Supplementary information). There are several scenarios proposed for this long relaxation process. First, it is generally assigned to the photo-voltage effect^29^. Moreover, huge Rashba-splitting effect has been clearly observed in BCB^30,31^, which might cause the long-time relaxation processes like indirect band-gap semiconductors^25^.

For p-type Sb_2_TeSe_2_, the Drude-SST-Kubo model is used to fit the results in Fig. 4f–i. It is worth emphasizing that the relative changes in *μ* and *T* are more distinct than the changes in *ω*_*p*_ and *Γ*. Even though the MIR probe-pulses can also detect FCA (even though it originates from TSSs or BSs as shown by the notation of probe(4) in Fig. 4j), the Δ*R*/*R* spectra are significantly dominated by SST’s in the Dirac cone (see the notations of probe(5) and probe(6)). Figure 4h shows that after the deep valance electrons are excited to the upper Dirac cone^5^, *μ* reaches a minimum at ~1 ps and it takes ~1.28 ps for the recombination process according to the fitting for a single exponential decay function. Besides, the hot-carrier temperature reaches ~600 K, and recovers to room temperature after 1.68 ps, which results are consistent with the time-resolved ARPES results for Sb_2_Te_3_^5–7^. Therefore, this hot-carrier temperature decay would be resulted from the thermal diffusion between BVB and TSS^27^.

By taking account of the difference of the number of states between bulk and surface, when the electrons are photo-excited, the chemical potential should shift towards the higher energy direction. In Drude-SST-Kubo model, the chemical potential (*μ*) and the carrier temperature (*T*) are associated with the surface state, and the SST-Kubo term is consisted by inter-transition and intra-transition of Dirac cone. For *μ* ≫ *T*, the intra-transition term could be derived to the form^20^ ${\varepsilon }_{F,intra}=\,-{e}^{2}|\mu |/\pi \hslash ({\omega }^{2}+i\omega {\varGamma }_{im})$ which coincides with the Drude expression, and the effective plasma frequency 𝜔_𝑝,S_ could be further expressed as $\sqrt{{e}^{2}|\mu |/\pi \hslash }$. More precisely, the contribution of excited charges to “𝜔_𝑝_” in Dirac cone is considered by the intra-transition term. In other words, the Drude term in Drude-SST-Kubo model represents the excited carriers which are out of the surface state. For p-type Sb_2_TeSe_2_ with the Fermi level locating at the lower energy part of the Dirac cone, after photoexcitation, the chemical potential shifts to the higher energy direction, and further indicates the redshift of plasma edge and decreasing of the density of states. From the fitting result of smaller 𝜔_𝑝_ (~1.3 × 10^3^ cm^−1^) on Sb_2_TeSe_2_, it shows the lower contribution from the excited bulk carriers, which is consistent with the results in Fig. 2b.

### Summary

The ultrafast dynamics of Dirac fermions and bulk free carriers in the TIs, n-type Bi_2_Te_2_Se and p-type Sb_2_TeSe_2_ single crystals, are studied using time-resolved ultra-broadband MIR spectroscopy. The dynamics in the n-type Bi_2_Te_2_Se is dominated by bulk carriers because the Δ*R*/*R* spectra show a blue-shift in the plasma edge due to FCA. For p-type Sb_2_TeSe_2,_ the dynamics is dominated by the Dirac fermion from the red-shift of the plasma edge in the Δ*R*/*R* spectra. This study shows that the MIR absorption peaks for FCA and SST in TIs can be distinguished and demonstrates the importance of time-resolved ultra-broadband MIR spectroscopy for gapless or small band gap exotic materials.

## Methods

### Experimental setup

Optical pump and ultra-broadband MIR probe spectroscopy^23^ consists of three stages: (i) 800-nm optical pulses with a duration of 30 fs were generated, (ii) ultra-broadband MIR probe pulses were generated in nitrogen and (iii) chirped pulses were generated for detection. The fundamental pulses (800 nm) and the second harmonic pulses (400 nm, which were generated by a type I *β-*BaB_2_O_4_ crystal with a thickness of 0.1 mm) from a Ti:sapphire amplifier (790 nm, 30 fs, 0.85 mJ at 1 kHz, Femtopower compactPro, FEMTOLASERS) were focused into nitrogen gas to generate MIR pulses. The filamentation occurred *via* four-wave DFG when the pulse was focused using a concave mirror (*r* = 1 m). The length of the filament was ~3 cm. The bandwidth and the duration of the generated MIR pulses were 200–5000 cm^−1^ and 8.2 fs, respectively. When the MIR pulses were reflected from the sample with an incident angle of 45°, they were converted to ~400-nm pulses for the detection using a chirped-pulse up conversion (CPU) in nitrogen gas. A third 800-nm beam was transmitted through dispersive materials, including four BK7 glass plates (thickness: 10 mm) and one ZnSe plate (thickness: 5 mm), to produce chirped pulses. The converted visible (VIS) spectrum was measured by a spectrometer with an electron-multiplying CCD camera (SP-2358 and ProEM+1600, Princeton Instruments). The time resolution was estimated to be ~60 fs. To prevent significant absorption from vapor, the system was placed in boxes whose interior was purged with nitrogen.

### Retrieving the MIR spectra from an up-converted spectra and calibrating the spectra of the VIS pulse to MIR region

The MIR spectrum form, especially the sharp absorption peaks, can be seriously distorted after CPU measurements. That is to say, the dispersion of chirped pulses causes additional oscillations in the spectrum^32,33^. The CPU signal (${E}_{{\rm{CP}}}^{2}(t-\tau ){E}_{{\rm{MIR}}}^{\ast }(t)$) was obtained by performing four-wave DFG (FWDFG, ${E}_{{\rm{FWM}}}(t)$) between the chirped pulse (${E}_{{\rm{CP}}}^{2}(t-\tau )$) and the MIR pulse (${E}_{{\rm{MIR}}}(t)$). The chirped pulse is written as:

3 $${E}_{{\rm{CP}}}(t)={ {\mathcal E} }_{{\rm{CP}}}(t){e}^{i{\omega }^{(0)}t+i\frac{1}{2}{\omega }^{(1)}{t}^{2}}$$

where ${ {\mathcal E} }_{{\rm{CP}}}(t)$ represents the envelope, ${\omega }^{(0)}$ is the central angular frequency, and ${\omega }^{(1)}$ is a chirp parameter. The MIR pulse can be divided into a main part ${E}_{{\rm{MIR}}}^{(0)}(t)$ and a free induction decay part ${E}_{{\rm{MIR}}}^{(1)}(t)$. Substituting ${E}_{{\rm{MIR}}}(t)={E}_{{\rm{MIR}}}^{(0)}(t)+{E}_{{\rm{MIR}}}^{(0)}(t)$ yields:

4 $${E}_{{\rm{FWM}}}(t)={E}_{{\rm{CP}}}^{2}(t){E}_{{\rm{MIR}}}^{(0)\ast }(t)+{E}_{{\rm{CP}}}^{2}(t){E}_{{\rm{MIR}}}^{(1)\ast }(t)={E}_{FWM}^{(0)}(t)+{E}_{FWM}^{(1)}(t)$$

where ${E}_{FWM}^{(0)}(t)$ can be assumed to be the Dirac delta function $\delta (t)$ due to the short duration of MIR pulse. Using the Wiener–Khinchin theorem and these assumptions, the autocorrelation ${C}_{{\rm{A}}}(t)$ of ${E}_{{\rm{FWM}}}(t)$ is formed by^33^

5 $$\begin{array}{ccc}{C}_{{\rm{A}}}(t) & = & {\int }^{}d{t}^{\text{'}}{E}_{{\rm{FWM}}}^{\ast }({t}^{\text{'}}){E}_{{\rm{FWM}}}({t}^{\text{'}}+t)\\  &  & =\delta (t)+{E}_{{\rm{MIR}}}^{(1)\ast }(t){ {\mathcal E} }_{{\rm{CP}}}^{2}(t){e}^{i2{\omega }^{(0)}t+i{\omega }^{(1)}{t}^{2}}\\  &  & +{E}_{{\rm{MIR}}}^{(1)}(-t){ {\mathcal E} }_{{\rm{CP}}}^{\ast 2}(-t){e}^{i2{\omega }^{(0)}t-i{\omega }^{(1)}{t}^{2}}\end{array}$$

A similar autocorrelation form ${C}_{{\rm{A}}}^{\text{'}}(t)$ is obtained for a pulse that is up-converted using a monochromatic pulse by multiplying ${e}^{-i{\omega }^{(1)}{t}^{2}{\rm{sign}}(t)}$, so that Eq. (5) becomes^33^

6 $${C}_{{\rm{A}}}^{\text{'}}(t)=\delta (t)+{E}_{{\rm{MIR}}}^{(1)\ast }(t){ {\mathcal E} }_{{\rm{CP}}}^{2}(t){e}^{i2{\omega }^{(0)}t}+{E}_{{\rm{MIR}}}^{(1)}(-t){ {\mathcal E} }_{{\rm{CP}}}^{\ast 2}(-t){e}^{i2{\omega }^{(0)}t}$$

Therefore, the original MIR spectrum with shift $2{\omega }^{(0)}t$ is acquired using the measured up-converted power spectrum and the known value of ${\omega }^{(1)}$ for the chirped pulse. Finally, the wavenumber is calibrated using a binomial fitting of the three absorption peaks, including carbon dioxide (~2300 cm^−1^) and water vapor (~1600 cm^−1^ and ~3700 cm^−1^).

### Analyses using the Drude, SST-Kubo and Drude-SST-Kubo models

In this study, the dielectric function $\varepsilon$ in the Drude model, the SST-Kubo model and the Drude-SST-Kubo model is used to calculate the p-polarized reflectivity *R*_*p*_ using the Fresnel equation (with an incident angle of 45°) as:

7 $${R}_{p}=\frac{\varepsilon \,\cos \,\theta -\sqrt{\varepsilon -{\sin }^{2}\theta \,}}{\varepsilon \,\cos \,\theta +\sqrt{\varepsilon -{\sin }^{2}\theta \,}}$$

The transient Δ*R*/*R* is obtained by:

8 $$\frac{\Delta R}{R}=\frac{{R}_{p}^{\ast }-{R}_{p}^{0}}{{R}_{p}^{0}}$$

where the superscripts “*” and “0” of *R*_*p*_ respectively represent the reflectivity with and without optical pumping. The fitting with the Drude model is performed using the software, RefFIT^34^. The fitting with the Drude-SST-Kubo model uses 4 parameters: ${\omega }_{p},\,\varGamma ,\,\mu$ and *T*. To limit the computational load without losing the accuracy, the grid search method and an interval search algorithm with few iterations are used. After obtaining all possible values for these 4 parameters, the most appropriate parameter set *P*^*j*^ is selected by calculating the minimum root-mean-square deviation between the data and the calculated results at the *j*^th^ iteration. More specifically, using the grid search method, the value of *P*^*j*^ at the *j*^th^ iteration can be obtained. The best interval is decided using the neighboring points of *P*^*j*^. In this analysis, 4 parameters produce the 8 neighboring points. Using this interval, the next iteration *j*+1 of the grid search is undertaken. Therefore, the accuracy is exponentially increased.

The conditions, ${R}_{p}^{0}$, are determined using the ARPES results and the FTIR spectra. For Bi_2_Te_2_Se, ${R}_{p}^{0}$ is calculated using the Drude model with ${\varepsilon }_{\infty }$ = 23.7, ${\omega }_{p}$ = 1880 cm^−1^ and *Γ* = 272 cm^−1^, which values are obtained by fitting the FTIR spectra using the RefFIT program^34^. For Sb_2_TeSe_2_, ${R}_{p}^{0}$ is determined using the Drude-SST-Kubo model with ${\varepsilon }_{\infty }$ = 19.4, ${\omega }_{p}$ = 1320 cm^−1^, *Γ* = 253 cm^−1^, *d*_*TSS*_ = 1.4 nm, *μ* = 72 meV and *T* = 297 K. The former 4 parameters are obtained by fitting with fixed values of *μ* and *T* using the grid search method and an interval search algorithm, as described previously. If *μ* is sufficiently large, it can be estimated as:

9 $$\mu =\sqrt{\pi {N}_{TSS}}\hslash {\upsilon }_{TSS}$$

where *N*_*TSS*_ is the surface carrier concentration (~2.2 × 10^12^ cm^−2^). The parameter *N*_*TSS*_ is expressed as:

10 $${N}_{TSS}=\frac{{A}_{FS}}{{A}_{BZ}{A}_{UC}}=\frac{\pi {K}_{F}^{2}}{\left(\frac{4{\pi }^{2}}{{a}^{2}}\right)({a}^{2})}=\frac{{K}_{F}^{2}}{4\pi }$$

where ${A}_{FS}$ is the area of the Fermi surface, ${A}_{BZ}$ is the area per Brillouin zone, ${A}_{UC}$ is the area per unit cell and *K*_*F*_ is the Fermi-wavenumber (~5.2 × 10^6^ cm^−1^ from ARPES). The parameter *υ*_*TSS*_ = 4.12 × 10^7^ cm/s is estimated from the gradient of Dirac cone from ARPES. More ARPES information of TIs is shown in S1 of Supplementary information.

### Exponential fitting in Fig. 4

The red line in Fig. 4d shows the bi-exponential fitting for *N*^(0)^+*N*^(1)^exp[−*t*/*τ*_*N*,1_]+*N*^(2)^exp[−*t*/*τ*_*N*,2_] for ${\omega }_{p}^{2}\,$(proportional to the time evolution of *N*) with a delay time *t*, where the parameters *N*^(0)^ = 4.16 × 10^7^ cm^−2^, *N*^(1)^ = 1.79 × 10^9^ cm^−2^, *N*^*(*2)^ = 2.6 × 10^8^ cm^−2^, *τ*_*N*,1_ = 1.5 ps, and *τ*_*N*,2_ = 8.4 ps. The red line in (h) shows the single-exponential fitting for *μ*^(0)^+*μ*^(1)^exp[-*t*/*τ*_*μ*_] for the transient chemical potential *μ*(*t*), where *μ*^(0)^ =72 meV is static chemical potential, *μ*^(1)^ is 99 meV and *τ*_*μ*_ = 1.28 ps. The red curve in Fig. 4i is fitted using a single-exponential function of *T*^(0)^+*T*^(1)^exp[−*t*/*τ*_*T*_] and the time evolution of the temperature, where *T*^(0)^ represents the room temperature, *T*^(1)^ is 770 K and *τ*_*T*_ is 1.68 ps.

## Supplementary information

Supplementary information.
