# Supplementary material for: Femtosecond time-evolution of mid-infrared spectral line shapes of Dirac fermions in topological insulators
Source: Sci Rep. 2020 Jun 17;10:9803. doi: 10.1038/s41598-020-66720-4 (PMC7299937; doi:10.1038/s41598-020-66720-4)
Supplement: Supplementary file 1 — Supplementary information. [file 41598_2020_66720_MOESM1_ESM.docx]

**Supplementary information for**

**Femtosecond time-evolution of** **mid-infrared** **spectral line shapes of Dirac fermions in topological insulators**

Tien-Tien Yeh,^1,2*^ Chien-Ming Tu,^1^ Wen-Hao Lin,^1^ Cheng-Maw Cheng,^2^ Wen-Yen Tzeng,^1^ Chen-Yu Chang,^1^ Hideto Shirai,^3^ Takao Fuji,^3,4^ Raman Sankar,^5,6^ Fang-Cheng Chou,^2,6^ Marin M. Gospodinov,^7^ Takayoshi Kobayashi,^1,8^ and Chih-Wei Luo^1,9,10*^

^1^*Department of Electrophysics, National Chiao Tung University, Hsinchu, Taiwan*

^2^*National Synchrotron Radiation Research Center, Hsinchu 30076, Taiwan*

^3^*Institute for Molecular Science, 38 Nishigonaka, Myodaiji, Okazaki 444-8585, Japan*

^4^*Toyota Technological Institute, 2-12-1 Hisakata, Tempaku-ku, Nagoya, 468-8511, Japan*

^5^*Institute of Physics, Academia Sinica, Nankang, Taipei R.O.C. Taiwan 11529*

^6^*Center for Condensed Matter Sciences, National Taiwan University, Taipei 10617, Taiwan*

^7^*Institute of Solid State Physics, Bulgarian Academy of Sciences, 1784, Sofia, Bulgaria, EU*

^8^*Brain science Inspired Life Support Research Center, The University of Electro-Communications, 1-5 1 Chofugaoka, Chofu, Tokyo 182-8585, Japan*

^9^*Taiwan Consortium of Emergent Crystalline Materials (TCECM), Ministry of Science and Technology, Taiwan*

^10^*Center for Emergent Functional Matter Science, National Chiao Tung University, Hsinchu 30010, Taiwan*

^*^E-mail address: [jiljiljilji@gmail.com](mailto:jiljiljilji@gmail.com) (T.-T. Yeh), [cwluo@mail.nctu.edu.tw](mailto:cwluo@mail.nctu.edu.tw) (C.-W. Luo)

**S1. Concentration and parameters of band structure of TIs:**

The topological surface and bulk carrier concentration are respectively given by ARPES and Hall measurement (van der Pauw method). From the ARPES result, the bulk state (BS) band gap *E_g_* of Bi_2_Te_2_Se is observed as ~0.30 eV. For p-type Sb_2_TeSe_2_, the minimum point of BCB occurs beyond *E_F_*, but BVB does not appear clearly as expected. An M-shaped resonant TSS was found instead of VB. The TSS concentration *N_TSS_* can be collected from Fermi wavenumber *K_F_* at Fermi level using the relation as Eq. (10) in manuscript. Besides, the chemical potential *μ* and Dirac fermion velocity *υ_TSS_* can be respectively estimated by Eq. (9) in manuscript and gradient of Dirac cone $\Delta E/\hbar\Delta k$. The following Table S1 demonstrates the parameters mentioned above.

In Fig. 1, the ARPES result exhibits that Sb_2_TeSe_2_ behaves as a p-type semiconductor with the Dirac point located above the Fermi level. Because the proposed band mapping result of Sb_2_TeSe_2_ in Fig. 1 was measured along the Γ-K direction, the valence band maximum in the middle of Γ-M direction was not seen in Fig. 1. The Dirac point was located above the Fermi level and could not probe by ARPES directly, but the existence of surface state in Sb_2_TeSe_2_ had been examined carefully by a photon energy dependent ARPES experiment and previous Shubnikov-de Haas oscillation experiment [1]. Because the TSS is protected by time-reversal symmetry, the existence of TSS is not changed by measurements in either ultra-high vacuum (UHV) or air. The robustness of topological protected TSS Sb_2_TeSe_2_ was examined previous studies [1-3].

**Table S1:** Parameters used for the fitting to the band structure in Fig. 1 with Eqs. (9) and (10).

| Sample | Dirac point | *K_F_* | *N_TSS_* | *υ_TSS_* | *μ* |
| --- | --- | --- | --- | --- | --- |
|  | (eV) | (×10^6^ cm^-1^) | (×10^12^ cm^-2^) | (×10^7^ cm∙s^-1^) | (meV) |
| Bi_2_Te_2_Se | -0.36 | 8.3 | 5.5 | 6.15 | 170 |
| Sb_2_TeSe_2_ | 0.19 | 5.2 | 2.2 | 4.14 | 70 |

**S2. “Quasi-equilibrium” state probed by a sub-10 fs pulse:**

In our time-resolved measurements, the probe pulse with a pulse duration of sub-10 fs is considered as a delta function because all of the observed time scale are extremely larger (> ps) than the pulse duration (< 10 fs). For each probe pulse, the measurement is in a view of “*relative equilibrium*” state or “*quasi-equilibrium*” state. Therefore, both of the Drude model and the Drude-SST-Kubo model could be further applied to the dynamical analysis with quasi-equilibrium state. For Drude model, although the states of excited carriers are non-equilibrium, the relaxation processes (> 1.5 ps for Bi_2_Te_2_Se and >1 ps for Sb_2_Te_2_Se) are hundreds of times larger than the pulse duration (< 10 fs) of a probe beam, so the state is considered as “*static*” during the moment of probe pulse measurements. For Drude-SST-Kubo model, the important issue is whether the excited states conform to the Fermi-Dirac distribution. According to the time-resolved ARPES [4], the excited carriers from deep valance band to shallow Fermi level is supposed to remain Fermi-Dirac distribution near the Fermi level, which is different from the excitation distribution of Bi_2_Te_2_Se [5,6] in Fig. S1, and can be detected by a probe beam. Hence, the Drude-SST-Kubo model is also adopted for the analyses of time-resolved measurements in this study.


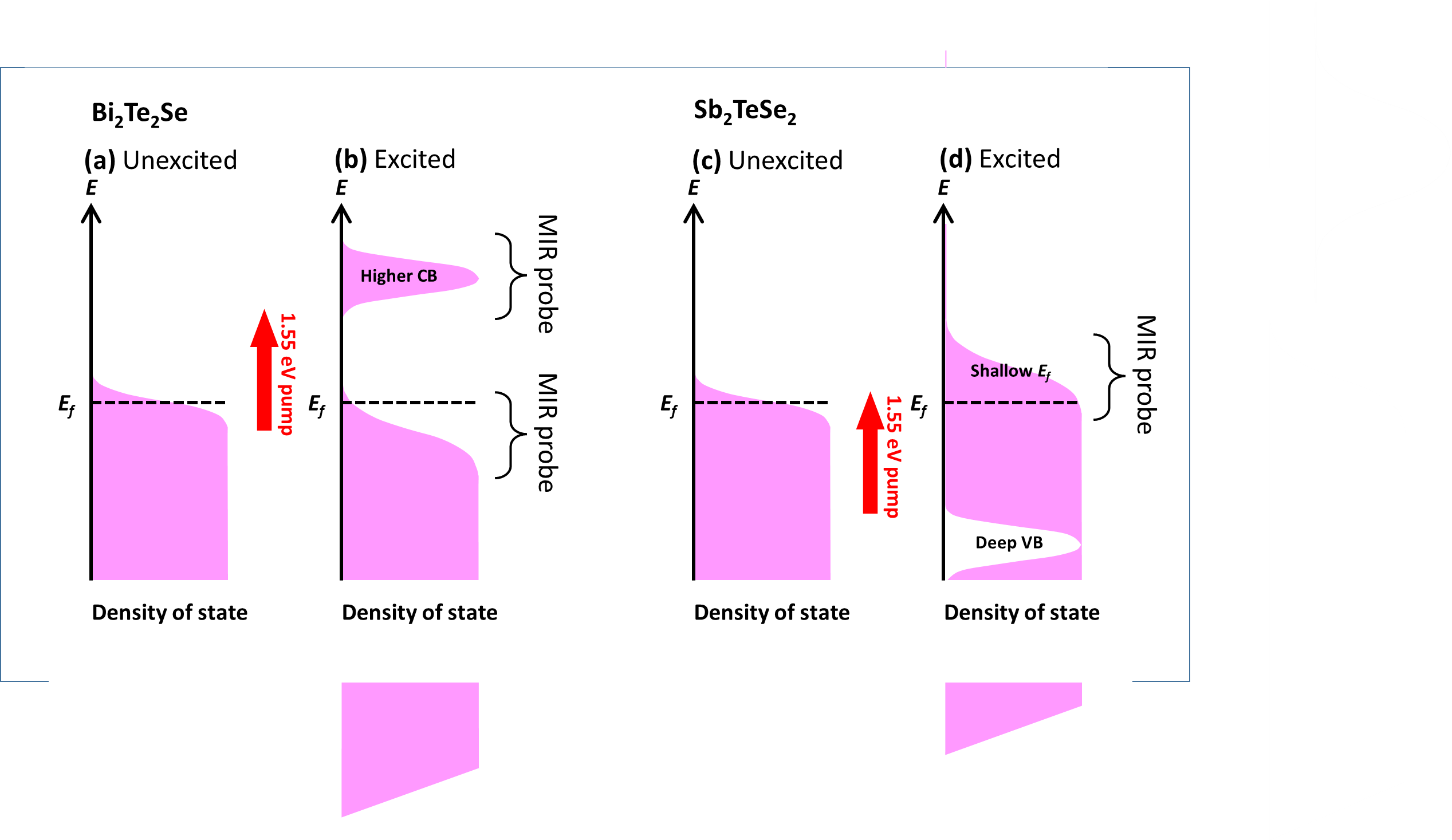


**Fig. S1.** The schematic of density of state before pump beam excitation (**a,c** for Bi_2_Te_2_Se, Sb_2_TeSe_2_ respectively) and after pump beam excitation (**b,d** for Bi_2_Te_2_Se, Sb_2_TeSe_2_ respectively). The notations marked by “MIR probe” indicate the possible areas for MIR probe detection.

**S3. Penetration depth of ultra-broadband MIR:**

The penetration depth of ultra-broadband MIR spectrum is demonstrated in Fig. S2 (~ few μm), which is larger than the penetration depth of 800 nm (~tens of nm [7]). Although the penetration depth of MIR probe beam covers the large parts of bulk, the photo-exciting depth caused by 800-nm pump beam leads to the dramatic change on surface. Therefore, the TSS signal is observable by MIR probe beam.

**Fig. S2**. Penetration depths of Bi_2_Te_2_Se and Sb_2_TeSe_2_ single crystals.

**S4. Concentration and parameters of band structure of TIs:**

The total incident phonon number of the 800-nm (1.55 eV) pump beam with fluence 101 μJ/cm^2^ is 4.0×10^26^ #/cm^2^. Notice that the penetration depth is the scale of nm, and we assume to be 20 nm [7]. Owing to the plasma frequency ~3.7 times lager (from 1880 cm^-1^ to ~7000 cm^-1^ after pump beam excitation), the carrier concentration is 13.9 times larger than the concentration before pumping. This experimental result indicates the excited concentration *N_ex_* become 1.7×10^20^ cm^-3^, which is expectedly smaller than the pump beam photon number. Furthermore, the quantum yield of 800-nm pump beam is estimated to 8.7×10^-7^.

**S5. Long-time relaxation process in Bi_2_Te_2_Se:**

To obtain the long-time relaxation process, we designed and built a system for transient reflectance measurement at the microsecond timescale as shown in Fig. S3a. We used 800 nm pulses (Ti:sapphire amplifier, 35 fs, 0.32 mJ at 5 kHz, Coherent: Legend) to pump the samples and measured the relaxation process using continuous IR light (water-cooled mercury lamp, Bruker). The mercury cadmium telluride (MCT) detector (J15D22-M204-S01M-60, Teledyne Judson Technologies​) with a time constant of 100 ns was used as an IR detector and was connected to an oscilloscope (GDS-1152A-U, GWInstek), which was synchronized with the laser for real-time data acquisition. Fig. S3b shows the reflected IR intensity as a function of time on the n-type Bi_2_Te_2_Se. The long relaxation time of 21.6 µs was unambiguously obtained by exponential fitting.

Up to date, there are several scenarios proposed for this long relaxation process. First, it is generally assigned to the photo-voltage effect although the results of Ref. [8] showed no photo-voltage effect on n-type TIs. Very recently, the photo-voltage effect was found to be enhanced by optical aging effect during long-time measurements and with larger pump fluence [9]. In this study, the samples were exposed to the amplified pump pulses for long time during pump-probe measurements. Consequently, the optical aging effect probably results in the long relaxation time of 21.6 µs in Fig. S3, which further causes the offset of carrier concentration (from $N_{unex}^{(0)}$ to $N^{(0)}$) in Fig. 4c and 4d of the manuscript. Alternatively, the appearance of bulk conduction band (see Fig. 1a in the manuscript) and band bending in Bi-based TIs may create the 2D quantum well state [9,10] to cause the long relaxation time (21.6 µs) of carrier concentration, which is similar to the results due to the surface photo-voltage effect.

Moreover, the long-time relaxation processes have been observed in indirect band-gap semiconductors, such as Ge [11]. Similar indirect band structure due to Rashba splitting was also reported for the long-time relaxation process in spin-orbit coupling materials [12,13]. For n-type Bi-based TIs, huge Rashba-splitting effect has been clearly observed in BCB [14,15]. For p-type Sb-based TIs, although the Rashba-splitting effect has been observed in BVB [2], our experimental results without the long-time relaxation process can be explained by DFT-Kubo model very well. More efforts will be necessary for a crucial determination, and we propose that temperature-dependent experiments may show more information for the underlying physics.


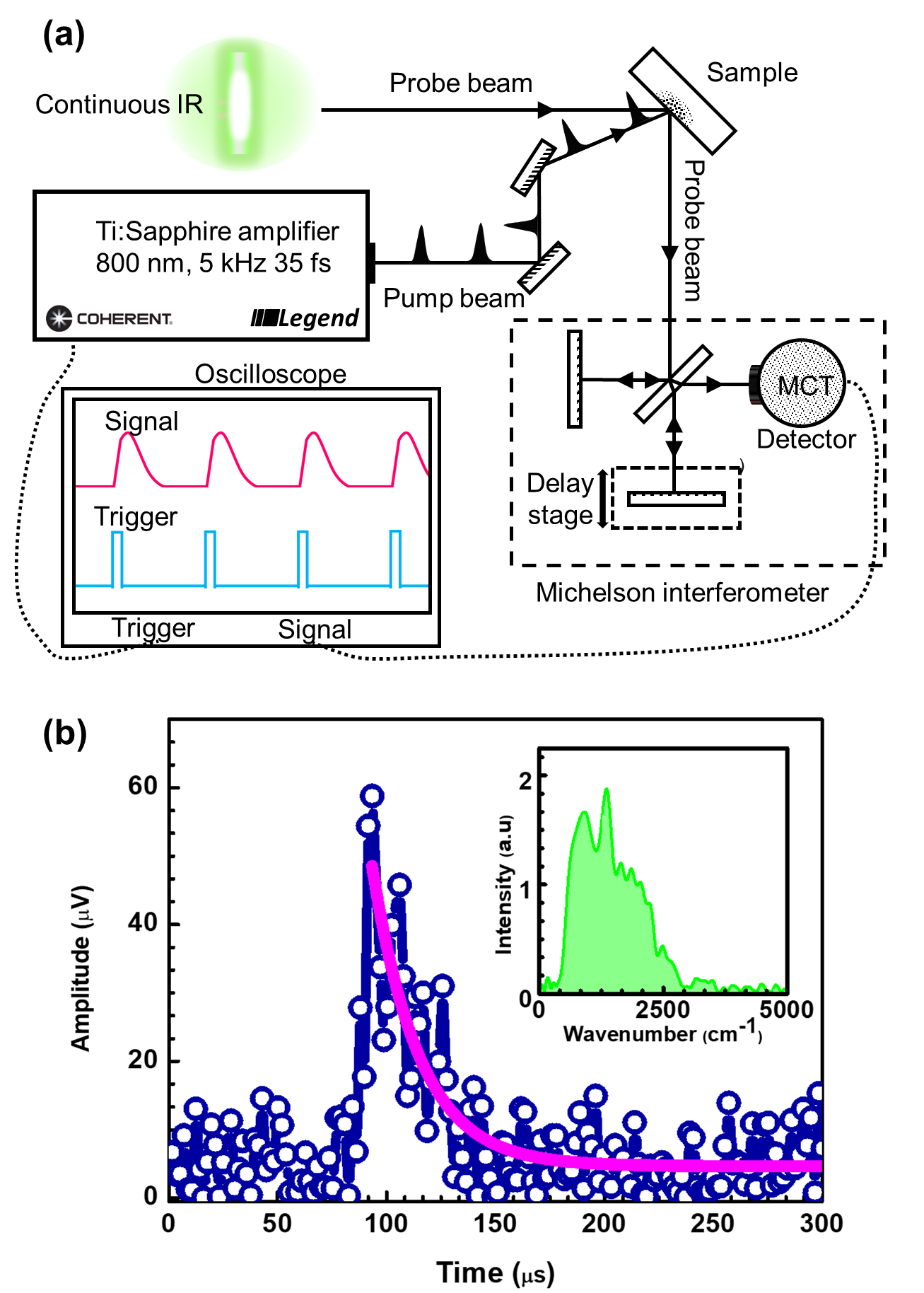


**Fig. S3**. **a** The scheme of 800-nm pulse pump and continuous IR probe system. **b** The reflected MIR intensity (blue circles) on Bi_2_Te_2_Se after pump pulse excitation with fluence of 100 µJ/cm^2^. The pink solid line shows the exponential fitting with the relaxation time of 21.6 µs. Inset: the Fourier transferred spectrum of IR probe beam, which measured by Michelson interferometer.

**References**

[1] Huang, S.-M. et al. Enhancement of carrier transport characteristic in the Sb_2_Se_2_Te topological insulators by N_2_ adsorption. *Sci. Rep.* **7**, 5133 (2017).

[2] Lee, C.-K. et al. Robustness of a Topologically Protected Surface State in a Sb_2_Te_2_Se Single Crystal. *Sci. Rep.* **6**, 36538 (2016).

[3] Fu, Y.-S. et al. Observation of Zeeman effect in topological surface state with distinct material dependence. *Nat. Comm.* **7**, 10829 (2016).

[4] Sánchez-Barriga, J. et al. Ultrafast spin-polarization control of Dirac fermions in topological insulators. *Phys. Rev. B* **93**, 155426 (2016).

[5] Wang, M. C., Qiao, S., Jiang, Z., Luo, S. N. & Qi, J. Unraveling photoinduced spin dynamics in the topological insulator Bi_2_Se_3_. *Phys. Rev. Lett.* **116**, 036601 (2016).

[6] Neupane, M. et al. Gigantic surface lifetime of an intrinsic topological insulator. *Phys. Rev. Lett.* **115**, 116801 (2015).

[7] Luo, C. W. et al. Snapshots of Dirac Fermions near the Dirac Point in Topological Insulators. *Nano Lett.* **13**, 5797−5802 (2013).

[8] Sterzi, A. et al. Bulk diffusive relaxation mechanisms in optically excited topological insulators. *Phys. Rev. B* **95**, 115431 (2017).

[9] Yoshikawa, T. et al. Enhanced photovoltage on the surface of topological insulator via optical aging. *Appl. Phys. Lett.* **112**, 192104 (2018).

[10] Chen, C. et al. Robustness of topological order and formation of quantum well states in topological insulators exposed to ambient environment. *PNAS* **109**, 3694-3698 (2012).

[11] Hall, R. N. Electron-Hole Recombination in Germanium. *Phys. Rev.* **87**, 387 (1952).

[12] Zheng, F., Tan, L. Z., Liu, S. & Rappe, A. M. Rashba Spin−Orbit Coupling Enhanced Carrier Lifetime in CH_3_NH_3_PbI_3_. *Nano Lett.* **15**, 7794−7800 (2015).

[13] Colton, J. S., Heeb, M. E., Schroeder, P., Stokes, A., Wienkes, L. R. & Bracker, A. S. Anomalous magnetic field dependence of the *T_1_* spin lifetime in a lightly doped GaAs sample. *Phys. Rev. B* **75**, 205201 (2007).

[14] Zhu, Z.-H. et al. Rashba spin-splitting control at the surface of the topological insulator Bi_2_Se_3_. *Phys. Rev. Lett.* **107**, 186405 (2011).

[15] Zhou, B. et al. Controlling the carriers of topological insulators by bulk and surface doping. *Semicond. Sci. Technol.* **27**, 12 (2012).
